# Role of water flow regime in the swimming behaviour and escape performance of a schooling fish

**DOI:** 10.1242/bio.031997

**Published:** 2018-09-20

**Authors:** Lauren E. Nadler, Shaun S. Killen, Paolo Domenici, Mark I. McCormick

**Affiliations:** 1 ARC Centre of Excellence for Coral Reef Studies, James Cook University, Townsville, Queensland 4811, Australia; 2 Department of Marine Biology and Aquaculture, James Cook University, Townsville, Queensland 4811, Australia; 3 Institute of Biodiversity, Animal Health and Comparative Medicine, University of Glasgow, Glasgow G12 8QQ, Scotland, United Kingdom; 4 CNR-IAMC, Istituto per l'Ambiente Marino Costiero, Localita Sa Mardini, Torregrande, 09170, Oristano, Italy

**Keywords:** Schooling behaviour, Fast-start behaviour, Anaerobic capacity, Habitat, Escape response, Plasticity

## Abstract

Animals are exposed to variable and rapidly changing environmental flow conditions, such as wind in terrestrial habitats and currents in aquatic systems. For fishes, previous work suggests that individuals exhibit flow-induced changes in aerobic swimming performance. Yet, no one has examined whether similar plasticity is found in fast-start escape responses, which are modulated by anaerobic swimming performance, sensory stimuli and neural control. In this study, we used fish from wild schools of the tropical damselfish *Chromis viridis* from shallow reefs surrounding Lizard Island in the Great Barrier Reef, Australia. The flow regime at each site was measured to ascertain differences in mean water flow speed and its temporal variability. Swimming and escape behaviour in fish schools were video-recorded in a laminar-flow swim tunnel. Though each school's swimming behaviour (i.e. alignment and cohesion) was not associated with local flow conditions, traits linked with fast-start performance (particularly turning rate and the distance travelled with the response) were significantly greater in individuals from high-flow habitats. This stronger performance may occur due to a number of mechanisms, such as an *in situ* training effect or greater selection pressure for faster performance phenotypes in areas with high flow speed.

This article has an associated First Person interview with the first author of the paper.

## Abstract (precis)

**Summary:** Fish exhibit flow-induced changes in their fast-start escape performance, likely due to an *in situ* training effect and/or selection for faster performance phenotypes in areas with high flow speed.

## INTRODUCTION

Environmental flow conditions (e.g. wind in terrestrial habitats and currents in aquatic systems) can be variable and rapidly changing in many habitat types (Madin et al., 2006; McLaren et al., 2014). For associated animal communities, variability in flow adds a level of complexity to activities such as foraging and navigation, particularly for animals that fly or swim (Krupczynski and Schuster, 2008; Riley et al., 1999; Srygley, 2001; Thorup et al., 2003). This additional challenge is the result of drift, in which animals must compensate for downstream displacement in order to effectively engage in essential activities (McLaren et al., 2014). In complex marine habitats, water flow patterns are influenced by wind, weather and tide conditions as well as the bathymetry of the benthos (Johansen, 2014; Madin et al., 2006; Nikora, 2010; Poff et al., 1997). In this era of rapidly changing climates, storm frequency and intensity are likely to increase in the future (Huntington, 2006), potentially changing temporal and spatial water flow patterns and breaking down structural complexity (Lilley and Schiel, 2006; Madin and Connolly, 2006). Acute high-flow events could present problems for animal assemblages, as the behaviour and physiology of resident animals are likely suited to their habitat's original conditions (Fulton and Bellwood, 2005; Johansen et al. 2007; Munks et al., 2015; Nunes et al., 2013).

Many fishes use group living (e.g. schooling) as a mechanism that may reduce energy costs associated with swimming (Abrahams and Colgan, 1985; Herskin and Steffensen, 1998; Marras et al., 2015; Weihs, 1973). Schooling is widespread among fish species and carries benefits for individuals with respect to predator avoidance, foraging opportunities and energy use (Krause and Ruxton, 2002; Nadler et al., 2016; Shaw, 1978). However, these benefits depend on how well the members of a school can coordinate their behaviours (Handegard et al., 2012). To maximise the benefits of grouping, schools exhibit plasticity in behavioural traits in response to individual needs and environmental stimuli, particularly in group cohesion, coordination and positional preferences (Hansen et al., 2015; Killen et al., 2012; Krause and Ruxton, 2002; Sogard and Olla, 1997; Ward and Webster, 2016; Webster et al., 2007). Environmental conditions such as water flow regime can influence behavioural and physiological phenotypes of both solitary and schooling fish (Anwar et al., 2016; Binning et al., 2015; Langerhans, 2008; Liao, 2007; West-Eberhard, 1989). Chicoli et al. (2014) found that individuals and schools exhibit a greater rate of reaction to a threat under an acute high flow treatment compared to a no-flow treatment. In wild-caught fish, Binning et al. (2014) found that fish from wave-exposed (and hence higher flow) sites exhibited greater aerobic swimming performance than individuals from sheltered (lower flow) sites.

The fast-start escape response is one of the main forms of defence used by fish against a predator that has initiated a strike. This response consists of a rapid, anaerobically-fuelled acceleration typically mediated by a pair of higher order command neurons called Mauthner cells (M-cell) in response to threatening sensory stimuli (Domenici, 2010; Korn and Faber, 2005). Hence, this behaviour is modulated by anaerobic swimming performance, sensory abilities and neural control. This type of response typically occurs on the order of milliseconds and is generally divided into three stages for the purpose of comparative analysis: stage 1 ­– unilateral muscle contraction on the side of the body opposite to the stimulus, causing the fish to bend into a C shape; stage 2 – contralateral muscle contraction, causing the tail to flip around creating additional forward acceleration; and stage 3 ­– variable stage with fish either gliding or burst swimming (Domenici and Blake, 1997; Tytell and Lauder, 2008; Wakeling, 2005). Anaerobic swimming performance in particular may be fundamental to survival under high flow conditions. Anaerobic swimming is characterised by burst-type swimming, powered by fast, glycolytic white muscle (Olson, 1998; Webb, 1998). These rapid movements may allow animals to cope with sudden changes in flow regime. In the absence of M-cell firing, escape responses can occur through activation of other homologous reticulospinal neurons, but typically exhibit a slower reaction time (i.e. latency) and kinematic output [e.g. turning rate in stage 1 and distance covered during the response; Eaton et al. (2001)]. Whether differences in the flow conditions experienced by an individual throughout development may alter fast-start escape responses of individual fish or fish schools through phenotypic plasticity or selection remains unknown (Binning et al., 2014, 2015).

Using schools of a gregarious coral reef fish, we investigated how native water flow regimes experienced at the school's home reef affected school swimming behaviour and individual escape performance. We hypothesized that, when tested under the same flow conditions in the laboratory, schools collected from higher flow habitats would exhibit more cohesive and coordinated (i.e. aligned) swimming behaviour. We expect this more effective swimming pattern to aid in eliciting faster and more agile escape responses in individuals from higher flow reefs, due to a combination of phenotypic plasticity to changing environmental conditions and stronger selective pressures in habitats with greater flow.

## RESULTS

**Fig. 1.**
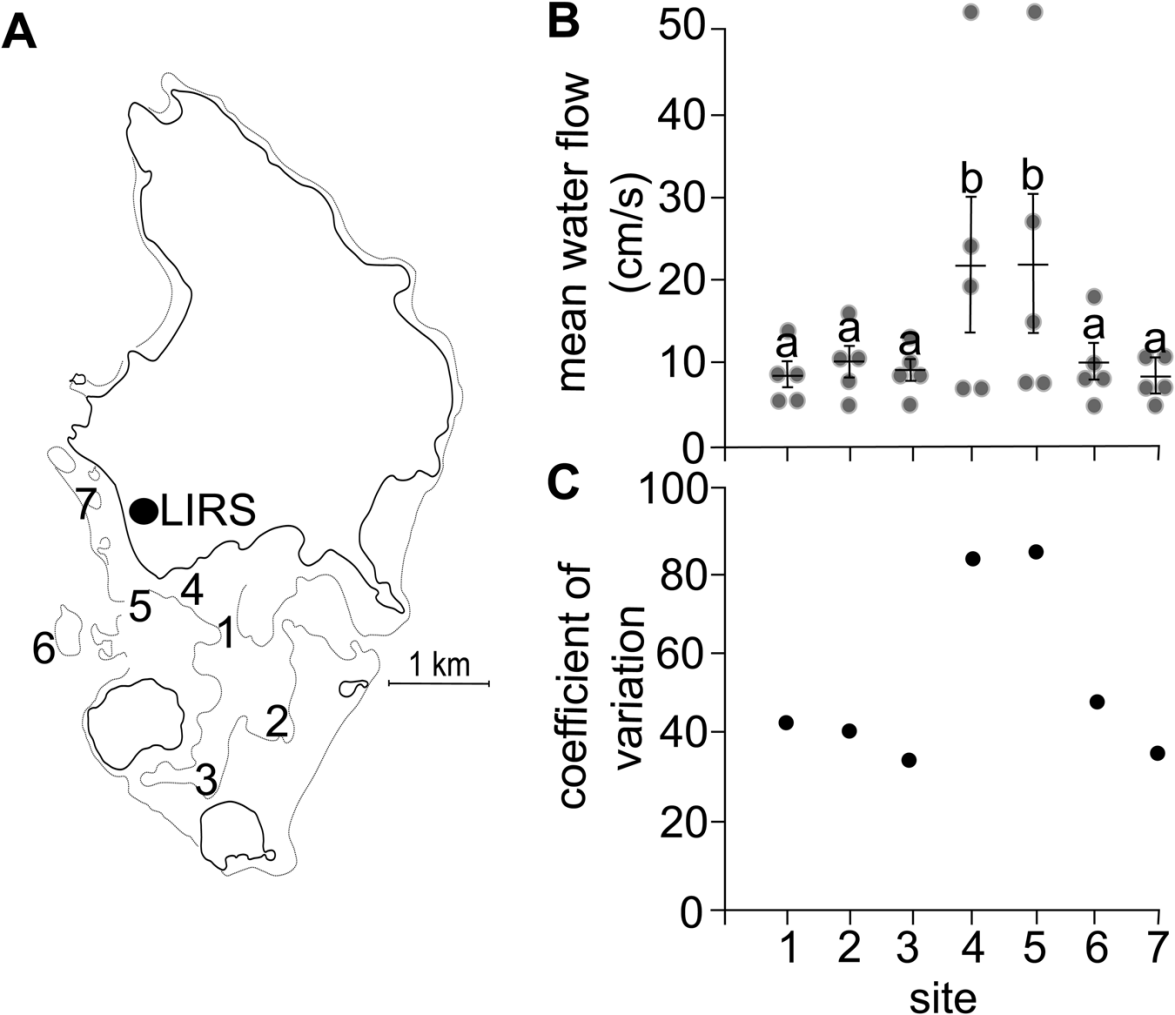
**Water flow speed.** (A) Map of the seven collection sites. (B) Mean water flow speed at each of the collection sites (±s.e.m.). Grey circles indicate values in 2 cm/s bins. (C) Coefficient of variation in water flow at each of the collection sites.

A subset of 11 wild schools of the tropical damselfish species *Chromis viridis* were collected from seven shallow reef sites in the Lizard Island lagoon, northern Great Barrier Reef, Australia (Fig. 1A). Water flow speed was measured on five separate days and differed significantly among sites (LMM: *F*_6,24_=3.35, *P*=0.0154; Fig. 1B). In particular, sites 4 and 5 exhibited a significantly higher water flow speed than sites 1, 2, 3, 6 and 7 (Tukey's test, *P*<0.05). In addition, flow speeds were more variable at sites 4 and 5 (Fig. 1C).

**Fig. 2.**
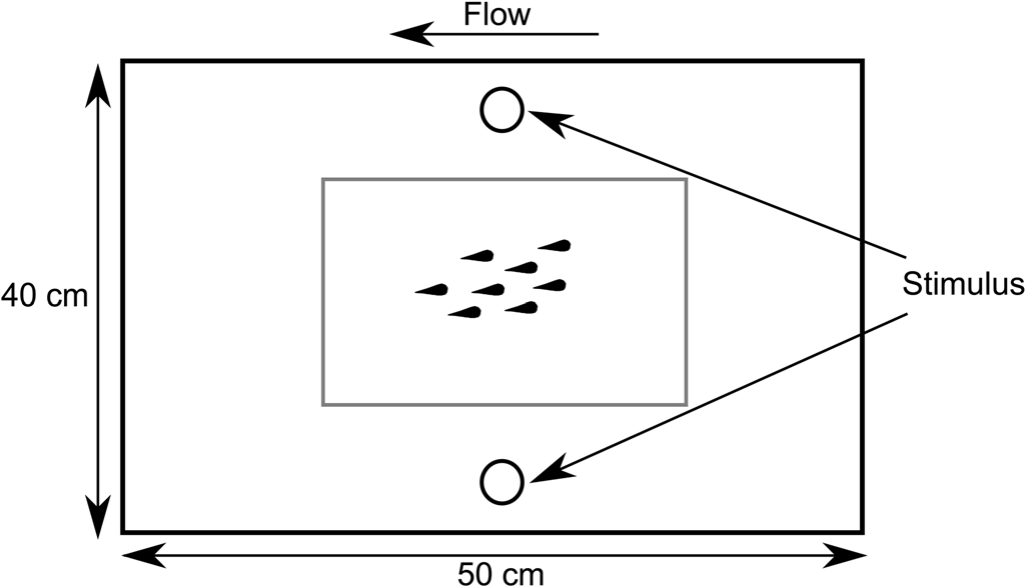
**Diagram illustrating the swim tunnel experimental arena.**

**Fig. 3.**
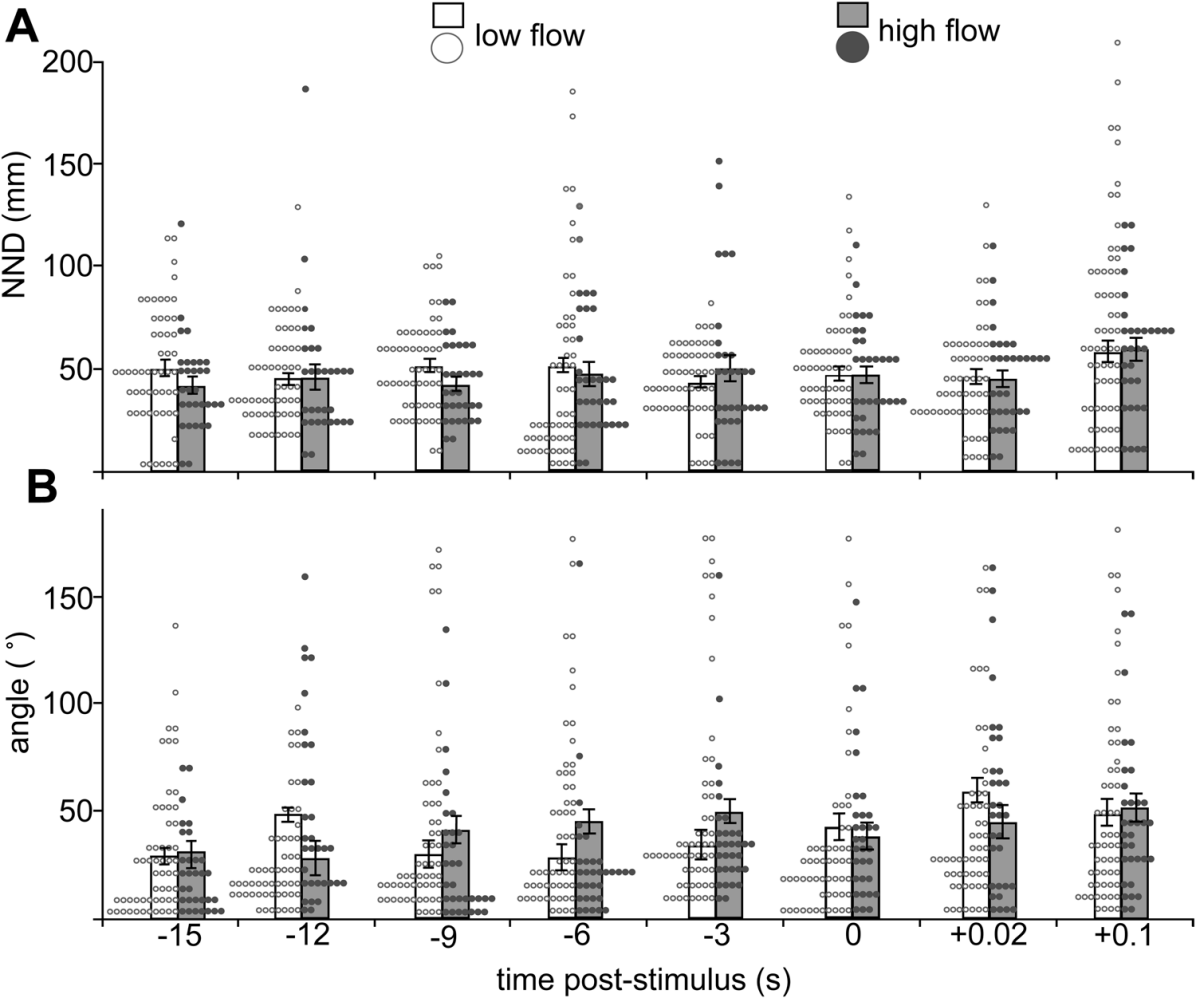
**School swimming performance.** (A) Nearest neighbour distance (NND) and (B) variability in individual alignment, before (−) and after (+) the stimulus. Bars are mean±s.e.m. Circles indicate data for individual fish in (A) 5 mm bins and (B) 5° bins. White circles and bars indicate data from low flow sites (*n*=56 fish) and grey circles and bars indicate data from high flow sites (*n*=32 fish).

School swimming and escape behaviour were assessed in a swim tunnel flowing at a slow speed [3.2 cm s^−1^ or ∼1 body length (*L*) s^−1^; Fig. 2] both before and after an aerial predator stimulus. *P*-values were corrected for type I error using a false detection rate (FDR) multiple test correction [p_cutoff_=0.0394; Benjamini and Hochberg (1995)]. Pre-stimulus behaviour was video-recorded at 30 frames per second (fps) and post-stimulus behaviour was recorded in high speed at 240 fps. School cohesion (as measured through nearest neighbour distance, NND) was not affected by either water flow speed from the school's home reef or time in relation to the stimulus [linear mixed-effects model (LMM) for flow: *F*_1,9_=0.21, *P*=0.6566; LMM for time: *F*_1,691_=1.42, *P*=0.2344; Fig. 3A]. School alignment was also not affected by water flow speed (LMM: *F*_1,9_=0.00, *P*=0.9891) but did vary significantly with time (LMM: *F*_1,691_=18.87, *P*<0.0001) (Fig. 3B). The interaction between water flow speed and time was not significant for either NND or alignment (*P*>p_cutoff_).

**Fig. 4.**
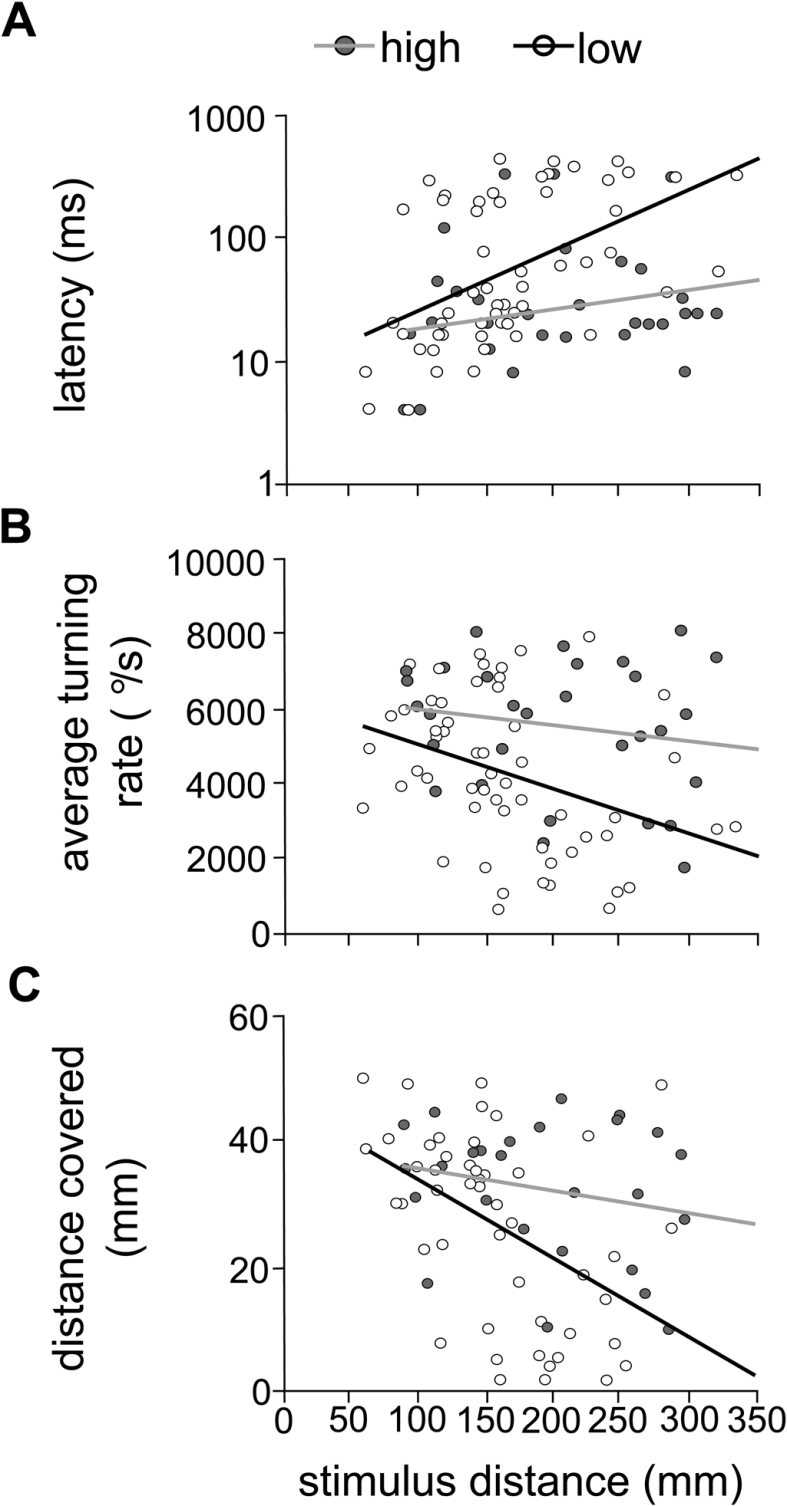
**Individual fast-start performance.** Individual fast-start performance according to high (grey dots with grey trend line, *n*=32 fish) or low (white dots with black trend line, *n*=56 fish) water flow regimes, including (A) latency (ms; log y-axis), (B) distance covered (mm) and (C) average turning rate (°/s).

Individual escape performance was assessed for latency, average turning rate during stage 1 of the reaction, and distance covered during the reaction (as a proxy for swimming speed). Individuals from high flow regime reefs exhibited greater escape performance, when compared with those collected from lower flow sites. There was a trend for latency in fish from high flow sites to be lower (indicating a faster reaction time) than in individuals from low flow sites, though this effect was not significant (Fig. 4A; LMM: *F*_1,5_=4.46, *P*=0.0883). In fish from higher flow sites, average turning rate was significantly greater than in fish from lower flow sites (Fig. 4B; LMM: *F*_1,5_=11.56, *P*=0.0193), suggesting that those fish from high flow sites exhibited a faster muscle contraction rate during stage 1 than those accustomed to lower flow. Distance covered was also significantly greater in fish from high flow sites than in fish from low flow sites (Fig. 4C; LMM: *F*_1,5_=7.67, *P*=0.0394), indicating that those fish swam a further distance with their escape response.

## DISCUSSION

Our study suggests that while school swimming behaviour is maintained across a range of localised water flow conditions, higher and more variable relative water flow speeds are associated with differences in individual fast-start performance. Water flow regime is a major driver in the distribution and abundance of behavioural, physiological and functional traits in fish assemblages in a range of systems (Binning et al., 2015; Fulton and Bellwood, 2005; McGuigan et al., 2003; Sinclair et al., 2014). As fast-start responses are modulated by a range of sensory and neural processes as well as anaerobic swimming performance, flow is likely inducing change on a range of functional levels within individual fish through plasticity and/or selection. These findings therefore indicate that an individual's performance may be tailored to the prevailing conditions at their home reef.

The effect of water flow speed on fast-start escape performance could occur as a result of a variety of mechanisms. Plasticity in behavioural traits in response to environmental factors has previously been demonstrated in response to water flow speed. Sinclair et al. (2014) observed increased boldness and aggression in the mosquitofish *Gambusia holbrooki* acclimated to a high flow environment when compared to those accustomed to low flow conditions. Any factor that increases the intensity and frequency of exercise in resident fishes may create a training effect that leads to improved physiological and behavioural performance (Anttila et al., 2011; Davison, 1997; Killen et al., 2016). A number of controlled laboratory studies have measured a training effect of water flow speed on aerobic metabolism and swimming performance, and found greater maximum metabolic rate, gait transition speed and critical swimming speed (Binning et al., 2015; Sinclair et al., 2014). Our results indicate that fast-start escape performance is likely subject to a similar training effect under high flow conditions, due to plasticity in anaerobic swimming performance, sensory systems and/or neural control. In humans, resistance training for both strength and endurance significantly improves anaerobic performance (Balabinis et al., 2003), suggesting that fish may also exhibit greater anaerobic swimming performance when they develop in high flow conditions. In addition, plasticity in the response to sensory cues occurs throughout ontogeny in a range of fish species in response to variable habitat conditions, by compensating for reduced cues in one sense with heightened sensitivity in other components of the sensory system (Chapman et al., 2010). Previous studies have also illustrated the scope for plasticity in fish neural activity, in M-cells in particular (Ebbesson and Braithwaite, 2012; Korn and Faber, 2005). Much of this plasticity in M-cell activity can be attributed to the varying sensitivity of neuromodulators to environmental conditions, which are integral in facilitating the transition from swimming to escape motor neurons (Song et al., 2015; Yeh et al., 1996). Variation in these neuromodulators may therefore be occurring in response to environmental flow conditions, consequently altering the escape neuron circuits' responsiveness.

Fast-start escape performance could also vary due to differences in selective pressure between high and low flow regimes (Higham et al., 2015). Previous studies have illustrated differential survival between fishes with varying locomotor performance (Swain, 1992). However, behavioural phenotypes may not experience a uniform degree of selective pressure across habitat types. For instance, slower performing individuals may experience stronger selective pressure in high flow compared to low flow regimes. This could account for the lower incidence of fish with ‘slow’ fast-start reactions than in schools collected from high flow regime reefs. In a study by Fu (2015), qingbo carp *Spinibarbus sinensis* exhibited a lower mortality rate when they had been acclimated to a high flow environment compared to those acclimated to still water. In addition, various studies suggest that water flow may reduce the ability of the lateral line to detect perturbations in the water created by attacking predators (Anwar et al., 2016; Feitl et al., 2010; Liao, 2006), potentially creating selection for individuals with a lower response threshold under high flow conditions. Further studies on how flow impacts predator strike performance and success would aid in understanding the contribution of selection to the distribution of fast-start phenotypes among habitat types.

Unlike fast-start performance of individual fish, there was no influence of water flow regime on school swimming behaviour. Previous studies have illustrated the plasticity of school cohesion and coordination in response to biotic and abiotic cues (Chivers et al., 1995; Cook et al., 2014; Domenici et al., 2002; Sogard and Olla, 1997; Webster et al., 2007; Weetman et al., 1998). Under an acute high flow treatment, Chicoli et al. (2014) found that schools of the giant danio *Devario aequipinnatus* exhibited increased alignment, orienting upstream into the flow. However, the results of this study suggest that this acute effect of flow on school structure does not translate into longer lasting effects on the school's behaviour when swimming at slower speeds.

Determining root causes of phenotypic divergence in wild-caught animals can be complicated by the difficulty in characterising all factors that may possibly influence results. Although we found differences in the relative water flow conditions among our study reefs, it is possible that additional factors that are correlated with water flow (e.g. predator density or behaviour, food availability, non-random sorting of individuals among groups) could be influencing the observed trends in escape behaviour (Fu et al., 2013; Killen et al., 2017; Marras and Domenici, 2013; Yan et al., 2015). Despite these potential disadvantages of characterising the distribution of behavioural phenotypes among animals from diverse natural environments, these types of studies are essential to place laboratory-based results in an ecological context and to understand real-world processes. Future work should aim to better understand which abiotic (e.g. turbulence) and biotic (e.g. food availability) factors associated with water flow drive the observed plasticity in escape behaviour. In addition, although it is potentially possible that the flow in the swim tunnel could affect the sensitivity of fish to mechano-acoustic stimuli, the flow in our set up was relatively slow (3.2 cm s^−1^). Therefore, any interference of the flow with the stimulation is likely to be minimal. We also controlled for the location of the school in reference to the stimulus (i.e. waited until six of the eight fish were in the box in the centre of the flow chamber) in order to minimise any effect of variability in school position on stimulus perception.

In complex habitats like coral reefs, changes in bathymetry due to disturbances such as storms can drastically alter flow conditions (Lilley and Schiel, 2006; Madin and Connolly, 2006). The results presented here suggest that a fish's defensive behaviour is tailored to its ambient environmental conditions. Therefore, it is possible that acute high flow events in coral reef habitats could create major challenges for fish assemblages in the future (Fulton and Bellwood, 2005; Munks et al., 2015; Nunes et al., 2013).

## MATERIALS AND METHODS

### Fish collection and maintenance

A subset of eleven wild schools of the tropical damselfish species *C. viridis* (standard length: 3.45±0.03 cm, body mass: 1.72±0.04 g, mean±s.e.) were collected from seven shallow reef sites (1.8–4 m depth; Fig. 1A) in the Lizard Island lagoon, northern Great Barrier Reef, Australia (14°40′08″S; 145°27′34″E). One to two schools were collected from each site, depending on the number of distinct schools found living on a given reef (not all sites contained multiple distinct schools, necessitating this design). Within sites, schools were separated by a minimum of 50 m and sites were separated by 400–3000 m. Fish were collected using hand nets and barrier nets and transported to the Lizard Island Research Station (LIRS). At the laboratory, fish were placed into experimental schools composed of eight individuals from their original schools and housed in replicate 20 l aquaria in a flow-through seawater system. Variation in body size within (<0.5 cm range) and among experimental schools (range=2.9–3.7 cm SL) was minimised (low flow average SL=3.31 cm, high flow average SL=3.47 cm).

*C. viridis* is an abundant, live coral-associated schooling species found on coral reefs throughout the Indo-Pacific region in groups ranging in size from three to hundreds of individuals (Nadler et al., 2014; Öhman et al., 1998; Pratchett et al., 2012). Information from a mark-recapture study indicated that *C. viridis* exhibit high site fidelity, with 64% of individuals found within 3 m of their home coral colony upon recapture three weeks later. Those individuals that had migrated >3 m from their home coral were found within an average of 34 m from their home coral colony, over a three-week period of calm weather (Nadler et al., unpublished data), suggesting that schools separated by a minimum of 50 m (as they were in this study) would be a part of distinct social groups. Fish were fed to satiation twice daily with INVE Aquaculture pellets and newly hatched *Artemia* sp. Laboratory tests of schooling characteristics and escape response were undertaken within seven days of capture, to avoid lab-induced changes in performance capacity.

### Water flow measurement

Water flow speed was measured at each of the seven collection sites on five separate days under varying wind and weather conditions, to determine relative differences in flow between sites. Measurements were always taken at high tide (±1 h). Flow speed was determined using a precision vane-wheel flow meter (Hontzsch Gmbh, Waiblingen, Germany) placed approximately 1.25 m below the water surface. As *C. viridis* forages on plankton in the water column above the reef throughout the day (Coughlin and Strickler, 1990; Smith and Warburton, 1992), this depth would be a realistic indicator of the flow conditions experienced by these schools during processes that require swimming (particularly foraging). Measures of flow speed (cm s^−1^) were logged at 1 Hz for 180 s. An overall mean flow speed was then calculated for each site using data from all five days.

A previous study at Lizard Island that found that water flow speed at shallow, sheltered reef sites (comparable to those used here) were dictated primarily by wind conditions. Particularly, sheltered sites that are <3 m in depth did not exhibit significant variability until the wind speeds exceeded 15 knots (Johansen, 2014). All measures of water flow speed were taken at <3 m depth and under wind speeds of <15 knots, allowing us to look at a relative measure of flow speed among our sites using this methodology.

### Swimming behaviour and escape response

Trials were conducted in a custom-built laminar flow swim tunnel (50 cm length×40 cm width×9 cm height; Fig. 2). This device allowed schools to swim in non-turbulent conditions at a slow uniform swim speed of approximately one *L* s^−1^ (3.2 cm s^−1^) for all trials, which mimics natural flow speed conditions at the seven collection sites on a calm day (Johansen, 2014). This low flow speed encouraged schools to swim but was slow enough that the fish were able to swim in any orientation to the flow (Nadler, personal observation). Seawater in the system was maintained at the ambient temperature for the study period (27–29°C). Experimental schools were placed in the swim tunnel and allowed to acclimate for 4 h. Afterwards, school swimming behaviour was video-recorded from below prior to the stimulus for 15 min (30 fps; Canon Powershot D10), using a mirror placed at a 45° angle. Escape responses were elicited using a standardised threat protocol in which a mechanical stimulus is dropped from above the experimental arena (Domenici et al., 2015). This stimulus was a black cylindrical object (2.5 cm diameter×12 cm length, 37.0 g) with a tapered end (to minimise surface waves), suspended 137 cm over the surface of the water in the swim tunnel. To avoid visual cues prior to the stimulus reaching the water's surface and to allow measuring response latency, this object was dropped through a white PVC pipe that ended immediately before it broke the water's surface (Domenici et al., 2008; Turesson and Domenici, 2007). A thread connecting the stimulus to the release point prevented it from touching the bottom of the tank (Domenici et al., 2008; Turesson and Domenici, 2007). As previous studies suggest that the school's alignment during an escape response is greatest with lateral stimulation at an angle of 30–120° (Marras et al., 2012), identical stimuli were placed 2 cm from each of the lateral walls in the centre of the swim tunnel. To control for a stimulus side preference, the use of the left or right lateral stimulus was alternated between trials. These stimuli remained suspended above the swim tunnel for the duration of the acclimation period using an electromagnet. Following the acclimation period, the stimulus was released using a switch, once a minimum of six of the eight fish were >3.5 cm from any wall of the swim tunnel and <4 L from the stimulus. This criterion aided in reducing the constraining effects that the walls of the swim tunnel may exert on an individuals' escape response and controlled for differences in escape performance that can occur with varying distance from the stimulus (Eaton and Emberley, 1991; Domenici and Batty, 1994, 1997). Each school's escape response was video-recorded from below in high speed (240fps; Casio Exilim HS EX-ZR1000). The swim tunnel was illuminated from above through a light diffusing filter using two 500 W spotlights.

### Kinematic analysis

Videos were analysed using the ImageJ software (v 1.42). School swimming behaviour before and after the stimulus as well as individual fast-start performance attributes were examined as defined below.

### School swimming behaviour

School swimming behaviour was characterised in terms of (1) school cohesion (nearest neighbour distance) and (2) alignment. (1) Nearest neighbour distance (NND): distance to the closest neighbour for each fish within the school, as measured by the distance from each fish’s centre of mass when stretched straight (CM). The location of the CM in video footage was measured as 0.35 L posterior of the snout, based on previous measurements of generalist fish species (Webb, 1978). The location of the fish’s CM when stretched straight is a useful point of measurement in fast-start studies on generalist fishes because of its strong ecological relevance for predator-prey interactions (Webb and Skadsen, 1980). (2) Alignment: the variation in the orientation of all school members to the horizontal (corresponding to the direction of flow; 180°, facing into the flow towards the front of the tank; 0°, oriented with the flow towards the back of the tank). As alignment angles spanned up to 360°, circular statistics were employed to find the school’s mean orientation (Bachelet, 1981), as calculated in the software Oriana 4 (Kovach Computing Services, Anglesey, Wales). In order to assess the alignment of each individual to their schoolmates’ orientation, alignment was calculated as the angular difference (in degrees) between each individual’s orientation and the school’s mean orientation. From the 15 min pre-stimulus video recording, five frames (one frame every 3 min for the duration of the recording) were analysed for each of the characteristics outlined below. In addition, school escape response variables were assessed at three times post-stimulus from the high-speed video recording (0, 20 and 100 ms post-stimulus). The stimulus onset was defined as the frame at which the stimulus first touched the water's surface (indicated by time=0 ms), and illustrates the school's behaviour immediately preceding stimulation. The remaining times (20 ms and 100 ms post-stimulus) were chosen because approximately one-third and two-thirds of fish in each school exhibited latencies for within each of those times respectively. Therefore, these times illustrate the school's behaviour early and late in the school's response to the threat stimulus.

### Individual fast-start performance

Individual escape performance was characterised through (1) response latency, (2) average turning rate and (3) distance covered. Previous studies suggest that the (4) stimulus distance can influence latency, average turning rate and distance covered (Domenici and Batty 1994, 1997), so this measure was included as a covariate in the analysis. (1) Response latency: the time period between the stimulus onset (contact with the water surface) and the fish's onset of the escape response. (2) Average turning rate: calculated by dividing the stage 1 angle [the angle between the lines intersecting the head and CM at the start and end of stage (1) by the duration of stage 1 (Domenici, 2004)]. Stage 1 is the stage immediately post-stimulus, in which fish contract the muscles on one side of their body, causing the fish to bend into a C shape. (3) Distance covered: distance that the fish's CM travelled within the first 10 frames (i.e. 42 ms) of their reaction. This duration was determined using a preliminary analysis, in which the average duration of stages 1 and 2 were calculated for the escape response of 24 individuals (i.e. one random fish per trial). This short time frame was used as a proxy for mean swimming speed in order to avoid issues with wall effects. Individuals less than 2 L from any wall of the swim tunnel at the time of their response were excluded from this analysis [10% of total; Eaton and Emberley (1991)]. (4) Stimulus distance: distance from the stimulus to the fish's CM.

### Statistical analysis

All statistical analyses were conducted in the R Statistical Environment v3.2.4 (R Development Core Team, 2016), using the packages ‘nlme’ and ‘multcomp’ (Pinheiro et al., 2016; Torsten et al., 2008). Residual and quantile-quantile plots were assessed for each model in order to ensure that all assumptions were met. To meet model assumptions, water flow speed and latency were log-transformed while NND and alignment were square-root transformed. Differences in water flow speed among sites were assessed using a linear mixed-effects model (LMM), with site as a fixed effect and sampling date as a random effect to account for differences in conditions among measuring days. For this analysis, flow was analysed as a continuous variable. Tukey's HSD post-hoc tests were used to further investigate significant differences between sites detected by the LMM.

For all remaining analyses, flow was analysed as a categorical variable (low flow=8.2–10 m/s; high flow=21.8 m/s). *P*-values were corrected for type I error using a false detection rate (FDR) multiple test correction [p_cutoff_=0.0394; Benjamini and Hochberg (1995)]. The influence of water flow speed on school swimming behaviour (NND and alignment) was tested using a LMM with water flow speed and time in relation to the stimulus (and their interaction) as fixed effects, with site, school and individual as random effects. Individual fast-start performance (latency, average turning rate and distance covered) was examined using a LMM with flow speed as a fixed effect and school and site as random effects. Stimulus distance was included as a covariate in this analysis. The R code used for this analysis has been included as electronic supplementary material.

## Supplementary Material

Supplementary information

First Person interview
