# Supplementary material for: Role of water flow regime in the swimming behaviour and escape performance of a schooling fish
Source: Biol Open. 2018 Sep 20;7(10):bio031997. doi: 10.1242/bio.031997 (PMC6215405; doi:10.1242/bio.031997)
Supplement: Supplementary information [file biolopen-7-031997-s1.pdf]

FIRST PERSON

First person – Lauren Nadler

First Person is a series of interviews with the first authors of a selection of papers published in Biology Open, helping early-career researchers promote themselves alongside their papers. Lauren Nadler is first author on 'Role of water flow regime in the swimming behaviour and escape performance of a schooling fish', published in BiO. Lauren conducted the research described in this article while a PhD student in Prof. Mark McCormick's lab at the ARC Centre of Excellence for Coral Reef Studies, Australia. She is now a postdoctoral research fellow in the labs of Prof. Ryan Hechinger at the Scripps Institution of Oceanography, USA, and Dr Øyvind Øverl at the Norwegian University of Life Sciences Norway. Her research examines links between animal behaviour and physiology, and assesses how these dynamics change with biotic (e.g., parasite infection) and abiotic (e.g., environmental disturbance) factors.

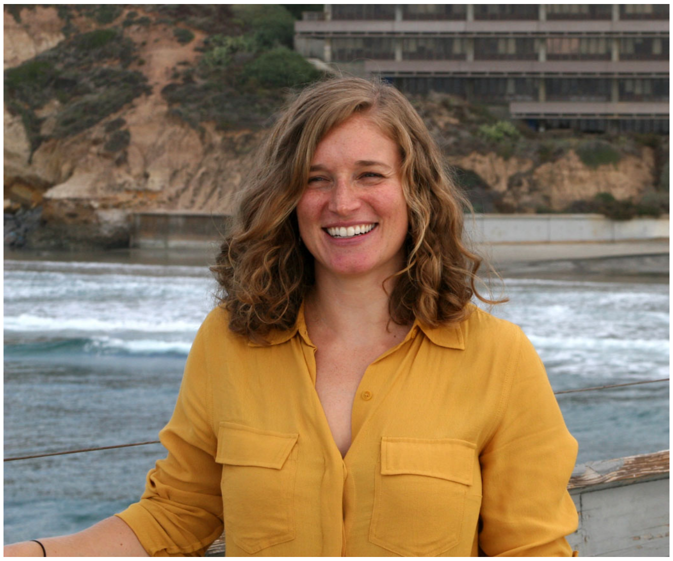

Lauren Nadler

What is your scientific background and the general focus of your lab?

I am a marine biologist, specialising in fish physiology and behaviour. Although I am originally from the USA, my scientific career has taken me around the world, from the Firth of Clyde in Scotland during my master's degree to the Great Barrier Reef in Australia for my PhD research. Fundamentally, my goal is to better understand why animals behave the way they do. That includes looking at both the animal itself as well as the environment the animal lives in. An individual's physiology can drive the behaviours that we see, but we are still very much learning how behavioural and physiological traits are connected and to what degree this drives individual variation within species. In addition, animals can tailor their behaviour to help them thrive where they live. We still have so much to learn on this topic, which I find really exciting.

How would you explain the main findings of your paper to non-scientific family and friends?

This study found that the way animals respond to threats can be tailored to the environment in which they live. To test this, we collected schools of fish from coral reefs with naturally low and high water flow rates, and measured how these fish responded to a simulated predator attack. The results indicate that those individuals that grew up in a fast-moving environment (high flow) had a much stronger escape performance, meaning that they would be more likely to escape a predator's attack than those from slower-moving habitats. This could be happening for a number of reasons. First, those individuals living in faster flow may be experiencing some sort of training effect, similar to humans that do high-intensity exercise regularly. Alternatively, slower performing individuals may just be less likely to survive in a fast-moving than in a slow-moving environment, so are simply eaten by predators first. The next steps will be to better understand why this is happening so that we can predict how environmental disturbances may shift the dynamics between predators and prey.

What has surprised you the most while conducting your research?

"No two days are ever the same, which keeps me excited to go to work on a daily basis."

I am always surprised by how creative this job is. As an experimental biologist, my job frequently requires me to think of new and innovative ways to measure an animal's characteristics in as realistic a setting as possible. This has required building a whole

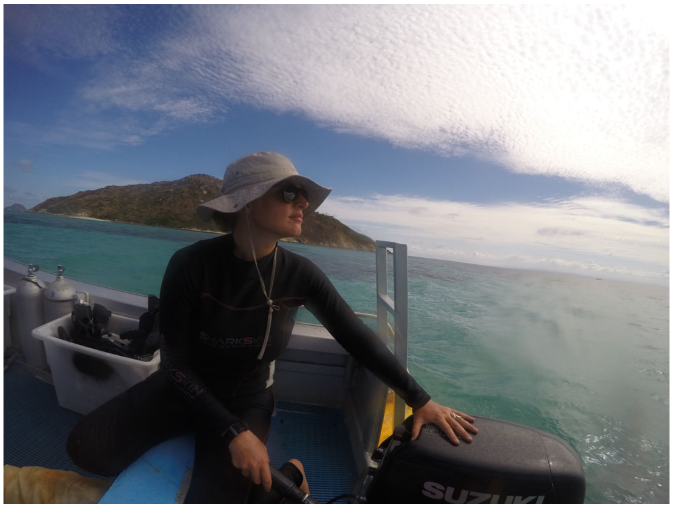

Dr Nadler heads out to the reef to collect schools of coral reef fish for her research at the Lizard Island Research Station, on the Great Barrier Reef, Australia.

Lauren Nadler's contact details: Norwegian University of Life Sciences, Ullevålsveien 72, Building 15, 0454 Oslo, Norway.
E-mail: lnadler@ucsd.edu

range of gadgets over the years, from a simulated flow tunnel for my fish to a treadmill for birds (a common fish predator). No two days are ever the same, which keeps me excited to go to work on a daily basis.

What's next for you?

Since finishing my PhD, I started a postdoctoral research fellowship based jointly between the Scripps Institution of Oceanography (USA) and the Norwegian University of Life Sciences (Norway), examining how parasites alter their hosts' behaviour and physiology in marine systems.

Reference

Nadler, L. E., Killen, S. S., Domenici, P. and McCormick, M. I. (2018). Role of water flow regime in the swimming behaviour and escape performance of a schooling fish. *Biol. Open* **7**, bio031997.
